# Antagonistic relationships between intron content and codon usage bias of genes in three mosquito species: functional and evolutionary implications

**DOI:** 10.1111/eva.12088

**Published:** 2013-07-24

**Authors:** Susanta K Behura, Brajendra K Singh, David W Severson

**Affiliations:** Eck Institute for Global Health, Department of Biological Sciences, University of Notre DameNotre Dame, IN, USA

**Keywords:** codon bias, coevolution, Intron, mosquito, ortholog

## Abstract

Genome biology of mosquitoes holds potential in developing knowledge-based control strategies against vectorborne diseases such as malaria, dengue, West Nile, and others. Although the genomes of three major vector mosquitoes have been sequenced, attempts to elucidate the relationship between intron and codon usage bias across species in phylogenetic contexts are limited. In this study, we investigated the relationship between intron content and codon bias of orthologous genes among three vector mosquito species. We found an antagonistic relationship between codon usage bias and the intron number of genes in each mosquito species. The pattern is further evident among the intronless and the intron-containing orthologous genes associated with either low or high codon bias among the three species. Furthermore, the covariance between codon bias and intron number has a directional component associated with the species phylogeny when compared with other nonmosquito insects. By applying a maximum likelihood–based continuous regression method, we show that codon bias and intron content of genes vary among the insects in a phylogeny-dependent manner, but with no evidence of adaptive radiation or species-specific adaptation. We discuss the functional and evolutionary significance of antagonistic relationships between intron content and codon bias.

## Introduction

Understanding functional and evolutionary patterns of insect genes and genomes has significant relevance to developing sustainable strategies for pest management, environmental protection, vector control, and disease prevention (Heckel 2003; Behura 2006; Grimmelikhuijzen et al. 2007; Gassmann et al. 2009; Tripet 2009; Severson and Behura 2012). Many human diseases including malaria, yellow fever, dengue fever, Japanese encephalitis, Rift Valley fever, Chikungunya, and West Nile are spread by different species of mosquitoes causing significant morbidity and mortality throughout the world (Fang 2010). However, not much is known on evolution of gene structures of mosquitoes that may have a role in the vectorial ability of mosquitoes to spread diseases. We are interested in codon bias and intron content of mosquito genes as our earlier study indicates that these features of *Aedes aegypti* genes are significantly associated with differential expression of genes in response to dengue virus infection (Behura and Severson 2012a). In addition, we have shown that codon bias patterns and expression patterns of *A. aegypti* and *Anopheles gambiae* genes are correlated (Behura and Severson 2011). Although a role of introns in gene function (particularly gene expression) is known (Castillo-Davis et al. 2002; Jeffares et al. 2008), it is unclear whether evolution of codon bias is related to intron sequences of genes among mosquito species.

Codon bias optimization or deoptimization are routinely used in biotechnological and biomedical applications in producing vaccines, recombinant products, and other beneficial products (Gustafsson et al. 2004, Fletcher et al. 2007, McArthur and Fong 2010, Mueller et al.*,* 2006; Coleman et al.*,* 2008). Also, studies show that codon bias patterns can be deoptimized (replacing optimal codons with rare synonymous codons) as a potential strategy to develop attenuated viral vaccines against specific viral diseases (Mueller et al. 2006, Coleman et al. 2008). Hence, understanding how introns may affect codon bias of genes is essential toward understanding functional evolution of mosquito genes and the potential applications thereof.

The origin and significance of introns in genes are not fully understood even three decades after their discovery (Gilbert 1978; Koonin 2006). Although studies of introns in several eukaryotes have provided useful insights into structure, function, and evolution of introns (Roy and Gilbert 2005a,b,c,d, 2006; Jeffares et al. 2006; Rodríguez-Trelles et al. 2006a; Li et al. 2009), the relationship of introns with codon bias is still an intriguing aspect of gene evolution in eukaryotes (Moriyama and Powell 1998; Vinogradov 2001; Fuglsang 2003; Le Hir et al. 2003; Chamary and Hurst 2005; Warnecke and Hurst 2007).

It is well established that codon usage bias, measured as nonrandom usage of synonymous codons, varies within and between species and has association with expression and translational selection of genes in prokaryotes as well as eukaryotes (Duret and Mouchiroud 1999; Carilini et al. 2001; Hershberg and Petrov 2008; Behura and Severson 2011, 2012b; Plotkin and Kudla 2011; Rodriguez et al. 2012). However, a clear evolutionary picture on the relationship between intron content and codon bias is lacking partly due to the fact that comprehensive analyses of codon bias and intron content of genes have been performed only in a few species, even though numerous eukaryote genome sequences are now available.

Understanding genome evolution of vector mosquitoes has been one of the major interests among vector biologists in recent times (Severson and Behura 2012). The genome sequences of three mosquitoes have been reported (Holt et al. 2002; Nene et al. 2007; Arensburger et al. 2010). These projects (https://www.vectorbase.org/) have provided new insights into the structure, function, and evolution of mosquito genes, furthering our ability to study mosquito–parasite or mosquito–virus interactions at the molecular level (Hill et al. 2005; Schneider and James 2006; Waterhouse et al. 2007, 2008; Behura et al. 2011). Although several studies have been performed on codon bias patterns in mosquito genomes (Behura and Severson 2011, 2012b; Rodriguez et al. 2012), no research has been conducted to understand how codon bias relates to intron loss or gain of genes in mosquitoes.

In this study, we investigated the relationship between codon bias and intron content in the orthologous genes of three important mosquito species: *Anopheles gambiae* (vector of malaria), *Aedes aegypti* (vector of dengue and yellow fever) and *Culex quinquefasciatus* (vector of lymphatic filariasis and West Nile virus) and then compared these relationships with more distantly related insects to obtain insights into the evolutionary links between codon bias and intron content of genes among the species.

## Materials and methods

The official gene sets annotated from whole genome sequences of *A. aegypti* (AaegL1.1), *C. quinquefasciatus* (CpipJ1), and *A. gambiae* (AgamP3.6) were obtained from VectorBase (http://www.vectorbase.org). The gene sequences were downloaded via the Biomart tool included in VectorBase. The official gene lists along with the coding sequences of three nonmosquito insect species *Drosophila melanogaster*, *Apis mellifera,* and *Pediculus humanus* were downloaded from either VectorBase or the ‘Ensembl Metazoa 10’ at http://www.biomart.org. The one-to-one orthologous genes either among the three mosquitoes or among all the six insect species (three mosquitoes and three nonmosquito species) were obtained from the Hierarchical Catalog of Orthologs (OrthoDB5) of arthropods (http://cegg.unige.ch/orthodb5).

The codon usage bias of the 1:1:1 orthologous genes (*n* = 6189) was determined by calculating the synonymous codon usage order (SCUO), effective number of codons (ENC), and codon adaptation index (CAI) as described earlier (Behura and Severson 2012b, 2013a). The intron counts, intron lengths, exon lengths, and gene lengths for each ortholog were determined from gene annotations of the corresponding species. Canonical correlation tests between codon bias indices (SCUO, ENC, and CAI) and intron contents (three measurements: number of introns, intron length relative to coding sequence length, and intron length relative to gene length) were performed using the ‘canonical analysis of principal coordinates’ method described in the study by Anderson and Willis (2003). This method of canonical correlation analysis was based on partitioning variation of the data into the principal coordinates, and then testing by permutation whether the variation was significant. First, a distance matrix was generated from the data based on the given variables that was then decomposed into its component eigenvalues and eigenvectors by principal coordinate analysis. The canonical axes scores (position of multivariate points on the canonical axes) were then used to determine correlations of each of the original variables with the canonical axes and to test by permutation whether the correlation was significant. These procedures were implemented in a FORTRAN computer program named ‘canonical analysis of principal coordinates (CAP)’ written by Dr Marti J. Anderson, University of Auckland.

Genes were classified as high intron content if intron counts were greater than the genome mean value of orthologous genes. The genome mean was calculated from the total counts of introns of the common genes (1:1:1 orthologous). Genes with intron numbers less than the genome mean value were classified as low intron content genes. Similarly, the mean SCUO value of the orthologous genes was calculated for each species, and genes associated with SCUO greater than the genome mean value of orthologous genes were classified as high biased, and those with SCUO less than the mean value were classified as low-biased genes. The count statistics of the four gene groups (genes associated with high or low codon bias and high or low intron content) were used to generate 2x2 contingency tables, which were subjected to Yates' chi square tests to determine statistically significant associations between intron content and codon bias in each genome. All statistical analyses were performed using the *R* statistical program. A *P*-value <0.05 was considered to be statistically significant in all tests unless stated otherwise.

The bootstrap randomizations of intron content and/or codon bias of the 1:1:1 orthologous genes were performed using MATLAB. Three independent randomizations were performed. In the first bootstrap experiment, codon bias and intron content values were simultaneously bootstrapped. Then, the frequency of the four gene groups [low (L)/high (H) intron content (INT) and/or low (L)/high (H) codon bias (CB)] was estimated. The bootstrap process was repeated 5000 times, and the mean values of the frequencies of the four gene groups were determined. In the second step, we bootstrapped only codon bias of genes for the same number of times (5000) while keeping intron content unaltered. In the third experiment, we bootstrapped the intron content of genes for 5000 times while keeping the codon bias values unaltered.

The 1:1:1 orthologous genes were classified as single exon (intronless) or multiexon (intron-containing) genes based on the number of exons of the annotated genes. Then, they were subcategorized into four groups based on low or high codon bias and intronless or intron-containing genes as described above. Binary logit regression was used to fit the data of codon usage bias (SCUO values as independent variables) of orthologous genes with the presence or absence of intron sequences in the genes (as dependent variables) among the three mosquito species. The dependent variable assumed a value 1 when the gene had intron(s), but 0 when it was intronless. The generalized linear model *y* = β_0_ + *X*β + e was used for the regression, where *y* = dependent variable (presence/absence of intron the gene), *X* = codon bias (SCUO value), β = coefficient of independent variable, and e (error) was assumed to be independent of *X* and had a standard logistic distribution with mean zero. The logit regressions were performed using the ‘glm’ (generalized linear model) function in *R*.

The hierarchical cluster analysis was performed among the orthologous genes (*n* = 226) across the six insect species (*A. gambiae*, *A. aegypti*, *C. quinquefasciatus*, *D. melanogaster*, *A. mellifera,* and *P*. *humanus*) based on average correlation method (de Hoon et al. 2004). The 226 one-to-one orthologous genes were identified among the six species using ‘single copy in all species’ search tool of OrthoDB database. The OrthoDB ID of orthology groups along with intron and codon bias data of genes of each species are listed in Table S1. A multi-Mantel procedure (developed by Dr. Liam J. Revell (http://anolis.oeb.harvard.edu/∼liam/programs/) was used to test significance of correlation of intron content with SCUO values of genes among species.

To further explore the associations of codon bias with intronless and intron-containing genes within and between species, a multifactorial analysis named ‘permanova’ (Anderson 2001) was used. permanova is based on partitioning of multivariate variation (defined by a distance measure) according to individual factors. The Euclidean distance was used as the measure of codon bias variation of intronless and intron-containing genes. First, the distances between each pair of observation units (sampling units) were calculated to obtain a distance matrix, which was then used to perform the test statistics according to the design of factors. Because the distribution of intronless or intron-containing genes was not uniform (*i.e.,* number of intronless or intron-containing orthologs vary between species), the codon bias of intronless and intron-containing orthologous genes among the three mosquitoes was randomized by methods as described in Camiolo et al. (2012). All randomizations (using MATLAB) were conducted 1000 times (100 independent randomizations each with sample size *n* = 10). No data transformation or standardization was done prior to analysis. Unrestricted permutation of the data was allowed to perform the permutation tests (4999 times) to determine the statistical significance of the analyses. While the *P*-value of significance of permanova tests were calculated based on *F*-ratio statistics, the significance of the pairwise *a posteriori* tests were based using the *t*-statistic (Anderson 2001). The *P*-value <0.05 was considered as significant association between codon bias and intron in each test, unless stated otherwise.

To investigate the variation between codon bias and intron content of genes in phylogenetic context, the ‘*BayesContinuous*’ program, developed by Pagel (1999), was used. The ‘B*ayesContinuous*’ method is useful for inferring trait correlation in relation to the species phylogeny (Pagel 1994, 1999). We examined a total of six insect species: *A. aegypti*, *C. quinquefasciatus*, *A. gambiae*, *D. melanogaster*, *A. mellifera,* and *P. humanus* in this analysis. These species were chosen because gene annotations (of the 226 genes mentioned above) of only these species allowed us to unambiguously determine codon bias and intron for each orthologous gene. All sequence alignments were performed by ClustalX (Larkin et al. 2007), and phylogenies were generated using *BayesPhylogeny* program (Pagel and Meade 2004). The phylogenies were rooted at *P. humanus* as it represented the most distant (phylogenetically) species among the 6 insects analyzed (Meusemann et al. 2010). The rooted phylogenies of the species were then analyzed by maximum likelihood methods implemented in ‘*BayesContinuous*’ program to detect any trend of directional evolutionary change between intron and codon bias. This was accomplished by testing two nested models: model A that corresponds to the standard constant-variance random walk model with a single parameter (representing instantaneous variance of evolution), and model B that predicts the variance of evolution parameter as in model A along with the directional change parameters, if any.

To characterize the directional covariance between codon bias and intron content across species, we included three scaling parameters into the model B. These scaling parameters (*kappa*, *lambda,* and *delta*) were estimated for both the traits simultaneously. The *kappa* parameter was used to test whether either punctuational or gradual modes of evolution existed between traits in a given phylogeny. The *delta* scale was used to detect whether the rate of covariance of traits had accelerated or slowed along the phylogeny, indicating adaptive radiation of the traits in species evolution. The parameter *lambda* reveals whether trait evolution was independent of species in the given phylogeny. All the scaling parameters were tested with maximum likelihood method of continuous regression with multiple maximum likelihood attempts (*n* = 10) per tree with no predefined restriction on regression coefficients. The likelihood ratio tests were carried out using the *R* statistical program.

## Results

### Comparison between codon bias and intron content

**Figure 1:**
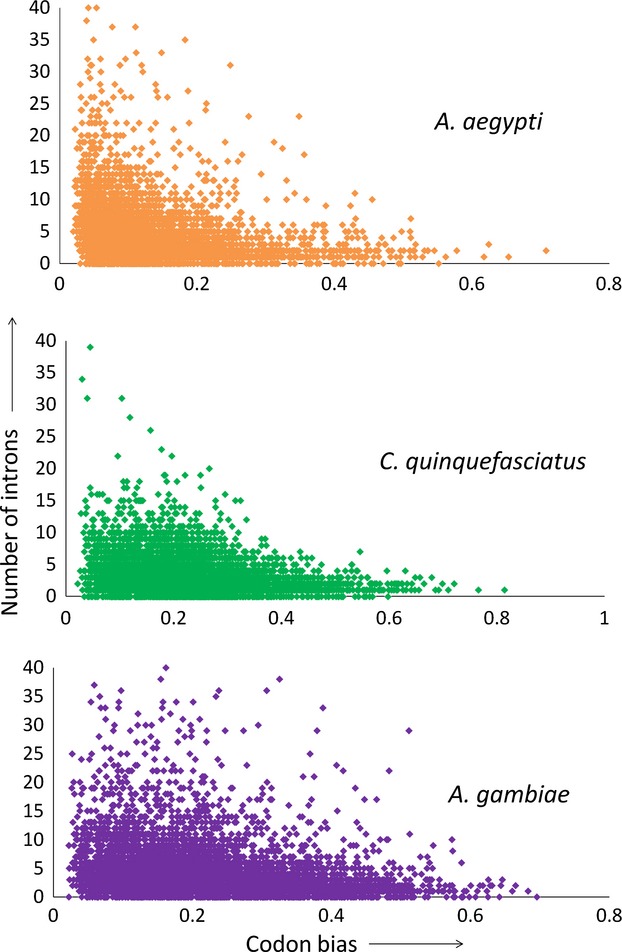
Scatter plots between codon bias and number of introns of 1:1:1 orthologous genes among *Aedes aegypti* (top), *Culex quinquefasciatus* (middle), and *Anopheles gambiae* (bottom). The *x*-axis represents codon bias index (SCUO), and *y*-axis represents number of introns of genes.

The 1:1:1 orthologous (*n* = 6189) genes among *A. gambiae*, *A. aegypti,* and *C. quinquefasciatus* were compared for intron content and codon usage bias. Different measures of codon bias of these genes such as synonymous codon usage order (SCUO), effective number of codons (ENC), and codon adaptation index (CAI) (see Behura and Severson 2013a for review) were compared with intron content of the corresponding genes in each species. Different measures of intron contents were introduced (i) the total number of introns of the gene, (ii) the total intron sequences per gene length (total intron length divided by gene length), and (iii) the total length of intron sequences per unit length of coding sequences of genes (total intron length divided by total exon length). The variations of the three measures of codon bias of the 6189 common genes were compared with the three measures of intron variation by canonical correlation method (Anderson and Willis 2003). The results showed the maximum correlation (the squared canonical correlation coefficient = −0.213) between SCUO and the total number of introns among the genes (Fig. 1). The comparison of SCUO with intron length among the orthologous genes is shown in Fig. S1. Similar distribution patterns were observed when SCUO values were compared with intron length normalized by total length of coding sequences (exons) or the entire gene length (data not shown). The correlation coefficients of SCUO with each of the three measures of introns are listed in Table S2. It shows that, although intron length and total counts of introns show similar distribution patterns of genes relative to SCUO, the magnitude of negative correlation between SCUO and intron length was less than the magnitude of correlation between SCUO and number of introns. Also, the negative correlation of ENC and CAI with the intron lengths showed lower magnitude than that of the correlation between SCUO and number of introns (data not shown). The eigenvalues of the principal axis of canonical variation between SCUO and all the three measures of intron content varied from 0.24 to 0.36 (Table S2). Based on permutation tests of the canonical correlations (Anderson and Willis 2003) among the 6189 orthologous genes, none of the correlations were statistically significant. However, when the orthologous genes were partitioned into gene groups varying in intron numbers and codon bias, significant association was evident by bootstrap analysis (see below). Nevertheless, in all cases, the overall correlation coefficient (among the all the orthologous genes) was found to be negative, indicating that intron and codon bias variation are antagonistically related to each other. As different codon bias measures revealed the same pattern of correlation between intron and codon bias variation, all subsequent analyses were performed with SCUO as the measure of codon bias. The choice of using this measure of codon bias was based on usefulness of SCUO measures in discriminating codon bias among different insect species (Behura and Severson 2012b).

**Figure 2:**
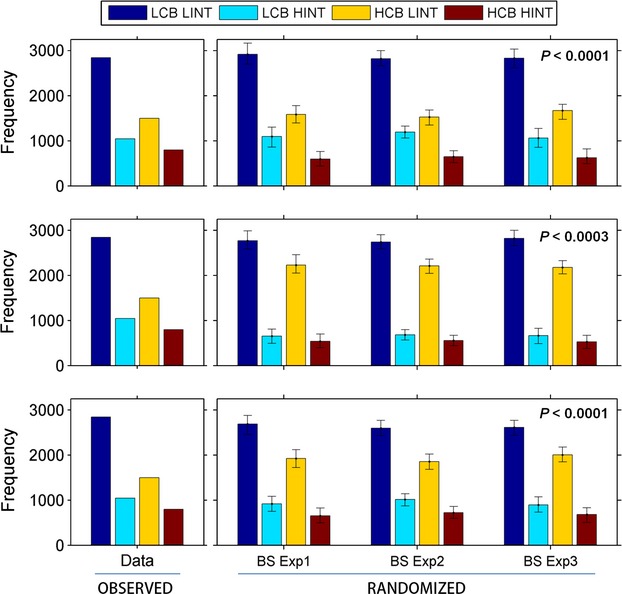
Number of genes associated with low (L)/high (H) intron number (INT) and/or low/high codon bias (CB) in *Aedes aegypti* (top row), *Anopheles gambiae* (middle row) and *Culex quinquefasciatus* (bottom row). The observed and the bootstrap experiment (BSExp) data are shown. In BSExp 1, both codon bias and intron number of the genes were randomized simultaneously, whereas either codon bias or intron number of the genes was randomized in BSExp 2 and BSExp 3, respectively. Error bars represent standard error values. The color code on the top of the graph represents the four gene groups. The abbreviation of these gene groups are as follows: LCB – low codon bias; HCB – high codon bias; LINT – low intron content; HINT – high intron content. The Yates' chi square *P*-values determined based on 2x2 contingency tests of the four gene groups, which remain unchanged between observed and randomized data sets, are shown for each species.

As the intron counts of genes revealed the greatest correlation with the codon bias (SCUO index) of the corresponding genes, it was hypothesized that the number of spliceosomal events in the gene may have a negative association with the extent of biased usage of synonymous codons. If that is true, then we expect a distribution pattern where genes with high codon bias as well as high intron numbers should be least frequent in the genome, and genes with low codon bias and low intron numbers should be most frequent. To test that the intron numbers of orthologous genes were plotted against the corresponding SCUO values of the three mosquitoes (Fig. 1). The results show that intron number peaks for genes within a narrow range of SCUO values (ca. 0.1–0.2), and then decreases as the codon bias increases. The mean SCUO value of the orthologous genes vary from 0.12 to 0.22, largely overlapping with the range where intron numbers of genes peak. The SCUO index varies from 0 to 1, with 0 representing genes with no codon bias and 1 representing genes with maximum codon bias (Wan et al. 2004). On the other hand, the mean intron count of the common orthologous genes was estimated at 2.3 in *A. gambiae*, 4.1 in *C. quinquefasciatus,* and 10.4 in *A. aegypti*. The number of genes associated with low/high codon bias and low/high intron number were determined based on the above mean values of codon bias and intron amount for each species. Yates' chi square tests reveal that the number of these genes vary significantly (*P* < 0.003) as shown in Fig. 2. To further ascertain that this observation is not due to random chance, we randomized the intron content and codon bias data in three different ways: (i) randomized codon bias across genes but keeping intron content unchanged, (ii) randomized intron content but keeping codon bias unchanged, and (iii) randomized both simultaneously. The results show that the randomized data assume similar distribution patterns between codon bias and introns as in the observed data (Fig. 2) suggesting that the antagonistic relationships between codon bias and intron content of genes represent nonrandom association in each genome.

### Intronless versus intron-containing genes and codon bias

**Figure 3:**
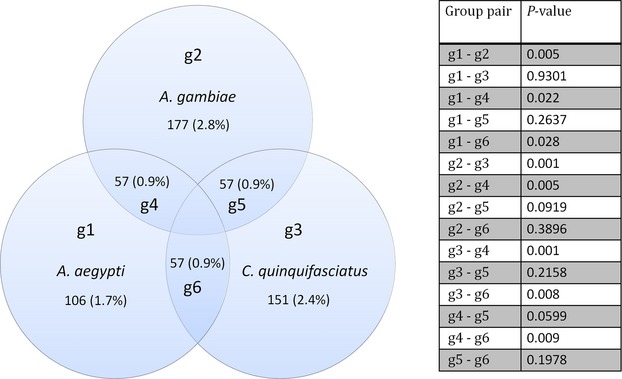
Distribution of intronless genes among the 1:1:1 orthologous genes among *Aedes aegypti*, *Culex quinquefasciatus,* and *Anopheles gambiae*. The Venn diagram shows the number and percentage of orthologous genes, which are intronless either within or between species. The significance of permanova tests between gene groups (g1 through g6) are shown to the right of the Venn diagram.

To know the relationship of intron presence or absence in the gene with the codon bias, the changes in SCUO of intronless and intron-containing 1:1:1 orthologous genes were compared among the three mosquitoes. The number of intronless and intron-containing orthologous genes that were associated with either lower or higher codon bias than the mean values shows significant (*P* < 0.002) variation in each species (Table 1). The data clearly show that the genes with low codon bias are predominantly intron containing, whereas genes of high codon bias are predominantly free of introns, further confirming that introns are antagonistic to codon usage bias of the genes. We performed pairwise *a posteriori* analysis of variance of codon bias among the orthologous gene groups (g1 through g6), which are either intronless or intron containing, as shown in Fig. 3. The permanova test (Anderson 2001) was conducted with an equal number of randomly sampled genes (*n* = 10) from each group for a total number of 100 independent randomization (see Methods). The purpose of data randomization was to mask possible confounding effects of distribution bias of introns in predicting association between intron and codon bias in the orthologous genes. The results of the multivariate analysis of variance revealed significant association (*F* = 3.72, df = 5 and *P* = 0.006) between codon bias and introns among different gene groups, as shown in Fig. 3. permanova was also used to perform pairwise *a posteriori* comparison between gene groups (see Fig. 3). This allowed us to determine the significance of intron vs. codon bias association within (*i.e.,* g1 *versus* g4, g2 *versus* g5 etc.) and between (*i.e.,* g1 *versus* g2, g2 *versus* g3, etc.) species. The results of these pairwise comparisons (Table S3) showed differential association of codon bias with the presence or absence of introns, as summarized in Fig. 3. However, it shows that only selective groups of genes significantly contribute to the antagonistic relationship between intron and codon bias. Hence, it is apparent that not all the common genes contribute to the observed antagonistic association between codon bias and introns, which may explain the lack of statistical significance of correlation between the two when compared among all the 6189 orthologous genes, mentioned above.

**Table 1:** Number of intronless (Intron-) and intron-containing (Intron+) genes associated with either low (L) or high (H) codon bias in three mosquito species. Chi square values and corresponding *P*-values of significance of association between codon bias and intron presence/absence are listed

	Intron-	Intron+	*P*-value
AaegL	115	5320	0.00165
AaegH	10	138	
AgamL	88	4838	4.65e–08
AgamH	37	620	
CquiL	98	4851	0.0008
CquiH	27	607	

Aaeg: *Aedes aegypti*; Agam: *Anopheles gambiae*; Cqui: *Culex quinquefasciatus*.

To further confirm the negative relationship of codon bias with intron content, binary logit regression was performed to fit the codon usage bias data with the distribution (presence or absence) of intron sequences of the orthologous genes among the three mosquito species. Based on the estimated regression coefficient of the logit model, it was observed that codon bias has differential marginal effects on the probability of the presence of introns of the 1:1:1 orthologous genes in each genome. The logit probability is given by 1/(1-e^(−*z*)^) where *z* = - [(codon bias of ortholog of *A. aegypti*)*0.31 + (codon bias of ortholog of *C*. *quinquefasciatus*)*0.27 + (codon bias of ortholog of *A. gambiae*)*4.48]. The negative coefficients of the regression analysis further confirm that codon bias has a negative association with the presence of intron sequence of genes of each species, although the magnitude of marginal effects of association varies among the species.

### Comparison with nonmosquito insect species

**Figure 4:**
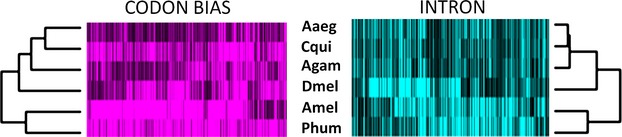
Hierarchical cluster patterns of codon bias (high: pink color and low: black color) and intron number (high: green color and low: black color) among orthologous genes (*n* = 226) among the three mosquitoes (*Aedes aegypti*, Aaeg; *Culex quinquefasciatus,* Cqui; and *Anopheles gambiae*, Agam) and three nonmosquito insects *Drosophila melanogaster*, Dmel; *Apis mellifera*, Amel; and *Pediculus humanus,* Phum). The cluster patterns of variation, in tree formats, are shown to the left and right of the corresponding self-organizing maps.

To further understand the relationship between introns and codon bias in a phylogenetic context, we extended the analysis to compare mosquito genes with orthologous genes of three distantly related insect species: *D. melanogaster* (fruit fly), *A. mellifera* (honeybee), and *P. humanus* (body louse). Hierarchical clustering between intron counts and codon bias was constructed for the orthologous genes (Table S1) among the three mosquitoes and three nonmosquito species (Fig. 4). Multi-Mantel test (Mantel 1967; Manly 2006) between codon bias and intron data matrix shows that there is a significant correlation (*r*^2^ = 0.72, *P* = 0.001) between intron and codon bias among the species.

Then, we wanted to know whether variation between codon bias and intron content is dependent upon the phylogenetic relationships among species. This was achieved by estimating likelihoods of two nested models, model A and model B (see Methods), of ‘*BayesContinuous*’ regression method (Pagel 1999). The intron number and codon bias values of 226 orthologous genes (Table S1) were used in the regression analysis simultaneously with the known phylogeny of the six species (three mosquito and three nonmosquito species). From the estimates of likelihood values of the two models, the log likelihood ratio test was conducted. Results suggested that codon bias and intron number data fit significantly better to model B than model A (log likelihood ratio = 12.3, *P* = 0.0004). This clearly indicates that the covariance between the two traits (codon bias and intron counts of orthologous genes) has a directional component associated with the species phylogeny. The estimated covariance between the two traits are always negative (Table S4), further confirming that codon bias and intron content covary antagonistically throughout the phylogeny.

To further characterize the directional nature of covariance between codon bias and intron number across species, we included three scaling parameters (*lambda*, *kappa,* and *delta*). Using *lambda* value as either 1 or 0 in the directional regression model, we observed that the log likelihood ratio test yielded significantly higher likelihood (log likelihood ratio = 12.3, *P* = 0.0001) for *lambda* = 1 compared to *lambda* = 0, suggesting that covariance between codon bias and intron is phylogeny dependent (*i.e.,* they coevolved according to phylogeny). Similarly, using the *kappa* parameter either equal to 0 (punctuational mode of evolution) or equal to 1 (gradual evolution), it was found that the likelihood of covariance between the two was significantly higher (log likelihood ratio = 9.9, *P* = 0.001) for gradual mode of evolution compared to punctuational mode of evolution, indicating that the coevolutionary relationship between codon bias and introns is a gradual evolutionary process in the phylogeny. The *delta* parameter was assigned values either greater than or less than 1 in the directional regression model, and the results showed no significant differences in the log likelihood ratio tests (data not shown). This indicated no evidence for accelerated or slower rates of covariance between codon bias and intron among the species.

## Discussion

The results obtained from this study provide insights into the evolutionary relationship between introns and codon bias of mosquito genes. It has been shown that unicellular organisms tend to have higher codon bias than multicellular organisms, particularly in highly expressed genes (Rocha 2004), and that the extent of codon usage bias of genes declines progressively with increasing generation time (Subramanian 2008) and genome complexity of the species (Biro 2008). At the same time, it is also argued that mean intron length of eukaryotes increases with genome size (Vinogradov 1999; Koonin 2006). If these trends are phylogeny dependent, the negative relationship between intron content and codon bias is likely to be manifested in mosquitoes and the other insects as we observed in the present study. Our result is consistent with the observation made by Vinogradov (2001) (Fig. S1), but provide more detailed investigation into the evolutionary aspects of the relationship in insects. In our analyses, the relative intron length was based on gene lengths from start to stop codons only. The untranslated regions (UTRs) of majority of the *A. aegypti* and *A. gambiae* genes have been predicted, but <15% genes of *C*. *quinquefasciatus* have been annotated for UTRs. Hence, to make an unbiased comparison, we used sequences from start to stop codon as the gene lengths in all the three mosquitoes.

The results from the current study further show that codon bias and intron content of genes covary among insect species as per the known phylogeny (Table S4). The association does not indicate any evidence of adaptive selection to any specific species or species groups. Thus, similar correlation was observed between intron content and codon bias across mosquitoes as well as the nonmosquito species. It is possible that GC content and mutation rates of genes may have a role in modulating correlated evolution of codon and intron sequences among the species. From our earlier study (Behura and Severson 2012b), we observed that while codon bias is favored by high GC content of dipteran genomes (such as mosquitoes), high AT content of genes favors biased usage of synonymous codons in the hymenopteran insects (such as honeybee). Furthermore, Haddrill et al. (2005) investigated the relationship between intron length and GC content of *Drosophila* genes and found a strongly negative correlation between intron length and rate of divergence of the genes. They found that such negative correlation was associated with local variation in mutational rates or biases in the genome. The mutational bias and GC content of gene sequences also explain differential number of introns and codon bias within mosquitoes. Within mosquitoes, the intron numbers also vary as follows: *A. gambiae* ∼38 000 introns, *A. aegypti* ∼51 000 introns, and *C. quinquefasciatus* 52 000 introns (Arensburger et al. 2010). At the same time, it is also known that *A. gambiae* has a relatively higher mutation rate (Waterhouse et al. 2007) but lower GC content (http://www.vectorbase.org) than *A. aegypti* and *C. quinquefasciatus*. This suggests that the lower amount of introns in *A. gambiae* than that of *A. aegypti* and *C. quinquefasciatus* may have an association with differential GC content and mutation rates of genes among these mosquitoes.

Links between gene expression and gene evolution have been suggested from several studies (Pál et al. 2001; Subramanian and Kumar 2004; Zhang and Li 2004; Drummond et al. 2005). According to these studies, genes that are less able to evolve are generally expressed at higher levels than genes that evolve faster. Moreover, it has also been shown that natural selection favors short introns in highly expressed genes (Castillo-Davis et al. 2002), and that intron-poor genes are regulated more efficiently for rapid changes in expression compared with that of intron-rich genes (Jeffares et al. 2008). These reports suggested that the presence of introns in the gene has a negative effect on its efficient expression and regulation. On the other hand, numerous studies have shown that codon bias is generally positively correlated with gene expression wherein the bias is usually high in highly expressed genes (Andersson and Kurland 1990; Rocha 2004; Behura et al. 2010; Behura and Severson 2011; Botzman and Margalit 2011). Based on these studies, it is possible that the negative relationship between codon bias and intron content may have a role in gene expression in eukaryotes.

There are also other possible reasons that may explain the negative relationship between intron and codon bias. The intron sequences in genes are known to constrain codon usage both near and away from the intron locations (Chamary and Hurst 2005; Parmley and Hurst 2007; Warnecke and Hurst 2007). This facilitates spliceosomal events to either increase intron numbers or decrease codon bias depending upon the favored codons in the species. Genes that are refractory to intron sequences are often expressed primarily in testes (Le et al. 2001), or are poorly transcribed (Rodríguez-Trelles et al. 2006b; Shabalina et al. 2010), or may be unrecognized pseudogenes (Zhu and Niu 2013), all of which may influence selection on codon bias. Several reports suggest that in many lineages, including some insects, intron loss dominates over intron gain (Roy and Gilbert 2005a, Carmel et al. 2007; Rogozin et al. 2012), indicating that intron number may also be determined in large part by rates of intron loss across genes. Given that intron loss may require reverse transcription of an mRNA transcript (Roy and Gilbert 2005d), this would suggest that highly expressed genes, which tend to have higher codon bias, would have fewer introns. Moreover, intron loss occurs preferentially in slowly evolving genes (Coulombe-Huntington and Majewski 2007a). These genes are generally associated with high codon bias (Davis and Petrov 2004; Yang et al. 2013). In mammalian genomes, it has been shown that intron loss occurs almost exclusively within highly expressed housekeeping genes, which are generally highly biased in codon usage (Coulombe-Huntington and Majewski 2007b). The role of intron loss in codon bias may be ubiquitous as antagonistic relationships between intron numbers and codon bias are also observed in plant genomes (Qin et al. 2013).

To put the results of our study into the broader evolutionary context, it is imperative to ask whether codon bias originated late or early in evolution. If we assume that codon bias might have an early evolutionary origin (Biro 2008), then the observed antagonistic relationship between codon bias and intron from our study would suggest that intronization of eukaryotic genes (Koonin 2006) may be associated with the decline of codon bias in higher organisms.

It should be noted that the genome sequences of the mosquitoes and also the other three nonmosquito insects that we used in the study do not represent the complete genome of the organism. The gene annotations are subject to quality of genome assembly and methods of gene model predictions. Although these factors have potential limitation to any genome-wide comparative study, it is unlikely that these limitations have any effect on the results of our current study. This is because we have limited our investigation to genes that have been annotated as one-to-one orthologs between species, and the chance that these genes are misannotated in all the genomes is remote.

Nevertheless, this is the first study aimed at understanding relationships of introns with codon bias of genes in mosquito species that spread deadly diseases to humans. It will provide opportunities for studying translational selection and its association with alternative splicing of genes in these species that constitutes a major gap in current knowledge of mosquito genomics (Severson and Behura 2012). Furthermore, codon usage bias is an important evolutionary factor of vector–pathogen interactions (Lobo et al. 2009). It has been demonstrated that intron content and codon bias have significant effects on gene expression in *A. aegypti* in response to dengue virus infection (Behura and Severson 2012a). In addition, codon bias and intracodon recombination also play a role in the evolution of the dengue virus that is transmitted by *A. aegypti* (Behura and Severson 2013b). Hence, our study may provide new directions for future studies aimed at better understanding the role of intrinsic features of genes, including that of intron content and codon bias, of mosquitoes in transmitting disease causing pathogens.

## Data archiving statement

Raw data for this manuscript are available in Dryad: doi:10.5061/dryad.qm44d.

## Supporting Information

**Table S1**. List of orthologous genes analyzed among the three mosquito species and three non-mosquito insect species (*Aedes aegypti*, *Culex quinquefaciatus*, *Anopheles gambiae*, *Drosophila melanogaster*, *Apis mellifera* and *Pediculus humanus*).

**Table S2.** Canonical correlations between synonymous codon usage orders (SCUO) and intron contents of 1:1:1 orthologous genes among three mosquito species.

**Table S3.** Results of pair-wise *a posteriori* comparison between gene groups (see Fig. 3).

**Table S4.** Summary results of the continuous regression tests. The beta parameter is the directional change parameter of model B.

**Figure S1**. Scatter plots between codon bias and intron length (normalized by exon length) of 1:1:1 orthologous genes among *Aedes aegypti* (top), *Culex quinquefasciatus* (middle) and *Anopheles gambiae* (bottom).

## References

[b1] Anderson MJ (2001). A new method for non-parametric multivariate analysis of variance. Austral Ecology.

[b2] Anderson MJ, Willis TJ (2003). Canonical analysis of principal coordinates: a useful method of constrained ordination for ecology. Ecology.

[b3] Andersson SG, Kurland CG (1990). Codon preferences in free-living microorganisms. Microbiological Reviews.

[b4] Arensburger P, Megy K, Waterhouse RM, Abrudan J, Amedeo P, Antelo B, Bartholomay L (2010). Sequencing of *Culex quinquefasciatus* establishes a platform for mosquito comparative genomics. Science.

[b5] Behura SK (2006). Molecular marker systems in insects: current trends and future avenues. Molecular Ecology.

[b6] Behura SK, Severson DW (2011). Coadaptation of isoacceptor tRNA genes and codon usage bias for translation efficiency in *Aedes aegypti* and *Anopheles gambiae*. Insect Molecular Biology.

[b7] Behura SK, Severson DW (2012a). Intrinsic features of *Aedes aegypti* genes affect transcriptional responsiveness of mosquito genes to dengue virus infection. Infection, Genetics and Evolution.

[b8] Behura SK, Severson DW (2012b). Comparative analysis of codon usage bias and codon context patterns between dipteran and hymenopteran sequenced genomes. PLoS ONE.

[b9] Behura SK, Severson DW (2013a). Codon usage bias: causative factors, quantification methods and genome-wide patterns: with emphasis on insect genomes. Biological Reviews.

[b10] Behura SK, Severson DW (2013b). Nucleotide substitutions in dengue virus serotypes from Asian and American countries: insights into intracodon recombination and purifying selection. BMC Microbiology.

[b11] Behura SK, Stanke M, Desjardins CA, Werren JH, Severson DW (2010). Comparative analysis of nuclear tRNA genes of *Nasonia vitripennis* and other arthropods, and relationships to codon usage bias. Insect Molecular Biology.

[b12] Behura SK, Gomez-Machorro C, Harker BW, Lovin B, de Bruyn DD, Hemme RR, Mori A (2011). Global cross-talk of genes of the mosquito *Aedes aegypti* in response to dengue virus infection. PLoS Neglected Tropical Diseases.

[b13] Biro JC (2008). Does codon bias have an evolutionary origin?. Theoretical Biology & Medical Modelling.

[b14] Botzman M, Margalit H (2011). Variation in global codon usage bias among prokaryotic organisms is associated with their lifestyles. Genome Biology.

[b15] Camiolo S, Farina L, Porceddu A (2012). The relation of codon bias to tissue-specific gene expression in *Arabidopsis thaliana*. Genetics.

[b16] Carilini DB, Chen Y, Stephan W (2001). The relationship between third-codon position nucleotide content, codon bias, mRNA secondary structure and gene expression in the drosophilid alcohol dehydrogenase genes *Adh* and *Adhr*. Genetics.

[b17] Carmel L, Wolf YI, Rogozin IB, Koonin EV (2007). Three distinct modes of intron dynamics in the evolution of eukaryotes. Genome Research.

[b18] Castillo-Davis CI, Mekhedov SL, Hartl DL, Koonin EV, Kondrashov FA (2002). Selection for short introns in highly expressed genes. Nature Genetics.

[b19] Chamary JV, Hurst LD (2005). Biased codon usage near intron-exon junctions: selection on splicing enhancers, splice-site recognition or something else?. Trends in Genetics.

[b81] Coleman JR, Papamichail D, Skiena S, Futcher B, Wimmer E, Mueller S (2008). Virus attenuation by genome-scale changes in codon pair bias. Science.

[b20] Coulombe-Huntington J, Majewski J (2007a). Intron loss and gain in *Drosophila*. Molecular Biology and Evolution.

[b21] Coulombe-Huntington J, Majewski J (2007b). Characterization of intron loss events in mammals. Genome Research.

[b22] Davis JC, Petrov DA (2004). Preferential duplication of conserved proteins in eukaryotic genomes. PLoS Biology.

[b23] Drummond DA, Bloom JD, Adami C, Wilke CO, Arnold FH (2005). Why highly expressed proteins evolve slowly. Proceedings of the National Academy of Sciences of the United States of America.

[b24] Duret L, Mouchiroud D (1999). Expression pattern and, surprisingly, gene length shape codon usage in *Caenorhabditis*
*Drosophila*, and *Arabidopsis*. Proceedings of the National Academy of Sciences of the United States of America.

[b25] Fang J (2010). Ecology: A world without mosquitoes. Nature.

[b82] Fletcher SP, Muto M, Mayfield SP (2007). Optimization of recombinant protein expression in the chloroplasts of green algae. Advances in Experimental Medicine and Biology.

[b26] Fuglsang A (2003). Patterns of context-dependent codon biases. Biochemical and Biophysical Research Communications.

[b27] Gassmann AJ, Onstad DW, Pittendrigh BR (2009). Evolutionary analysis of herbivorous insects in natural and agricultural environments. Pest Management Science.

[b28] Gilbert W (1978). Why genes in pieces. Nature.

[b29] Grimmelikhuijzen CJ, Cazzamali G, Williamson M, Hauser F (2007). The promise of insect genomics. Pest Management Science.

[b83] Gustafsson C, Govindarajan S, Minshull J (2004). Codon bias and heterologous protein expression. Trends in Biotechnology.

[b30] Haddrill PR, Charlesworth B, Halligan DL, Andolfatto P (2005). Patterns of intron sequence evolution in *Drosophila* are dependent upon length and GC content. Genome Biology.

[b31] Heckel DG (2003). Genomics in pure and applied entomology. Annual Review of Entomology.

[b32] Hershberg R, Petrov DA (2008). Selection on codon bias. Annual review of genetics.

[b33] Hill CA, Kafatos FC, Stansfield SK, Collins FH (2005). Arthropod-borne diseases: vector control in the genomics era. Nature Reviews Microbiology.

[b34] Holt RA, Subramanian GM, Halpern A, Sutton GG, Charlab R, Nusskern DR, Wincker P (2002). The genome sequence of the malaria mosquito *Anopheles gambiae*. Science.

[b35] de Hoon MJL, Imoto S, Nolan J, Miyano S (2004). Open source clustering software. Bioinformatics.

[b36] Jeffares DC, Mourier T, Penny D (2006). The biology of intron gain and loss. Trends in Genetics.

[b37] Jeffares DC, Penkett CJ, Bähler J (2008). Rapidly regulated genes are intron poor. Trends in Genetics.

[b38] Koonin EV (2006). The origin of introns and their role in eukaryogenesis: a compromise solution to the introns-early versus introns-late debate?. Biology Direct.

[b39] Larkin MA, Blackshields G, Brown NP, Chenna R, McGettigan PA, McWilliam H, Valentin F (2007). ClustalW and ClustalX version 2.0. Bioinformatics.

[b40] Le Hir H, Nott A, Moore MJ (2003). How introns influence and enhance eukaryotic gene expression. Trends in Biochemical Sciences.

[b41] Le YJ, Kim H, Chung JH, Lee Y (2001). Testis-specific expression of an intronless gene encoding a human poly(A) polymerase. Molecules and Cells.

[b42] Li W, Tucker AE, Sung W, Thomas WK, Lynch M (2009). Extensive, recent intron gains in *Daphnia* populations. Science.

[b43] Lobo FP, Mota BE, Pena SD, Azevedo V, Macedo AM, Tauch A, Machado CR (2009). Virus-host coevolution: common patterns of nucleotide motif usage in Flaviviridae and their hosts. PLoS ONE.

[b44] Manly BFJ (2006). Randomization, Bootstrap and Monte Carlo Methods in Biology.

[b45] Mantel N (1967). The detection of disease clustering and a generalized regression approach. Cancer Research.

[b84] McArthur GH, Fong SS (2010). Toward engineering synthetic microbial metabolism. Journal of Biomedicine & Biotechnology.

[b46] Meusemann K, Simon BM, von Reumont S, Roeding F, Strauss S, Kück P, Ebersberger I (2010). A phylogenomic approach to resolve the arthropod tree of life. Molecular Biology and Evolution.

[b47] Moriyama EN, Powell JR (1998). Gene length and codon usage bias in *Drosophila melanogaster*
*Saccharomyces cerevisiae* and *Escherichia coli*. Nucleic Acids Research.

[b85] Mueller S, Papamichail D, Coleman JR, Skiena S, Wimmer E (2006). Reduction of the rate of poliovirus protein synthesis through large-scale codon deoptimization causes attenuation of viral virulence by lowering specific infectivity. Journal of Virology.

[b48] Nene V, Wortman JR, Lawson D, Haas B, Kodira C, Tu ZJ, Loftus B (2007). Genome sequence of *Aedes aegypti*, a major arbovirus vector. Science.

[b49] Pagel M (1994). Detecting correlated evolution on phylogenies: a general method for the comparative analysis of discrete characters. Proceedings of the Royal Society of London. Series B: Biological Sciences.

[b50] Pagel M (1999). The maximum likelihood approach to reconstructing ancestral character states of discrete characters on phylogenies. Systematic Biology.

[b51] Pagel M, Meade A (2004). A phylogenetic mixture model for detecting pattern-heterogeneity in gene sequence or character-state data. Systematic Biology.

[b52] Pál C, Papp B, Hurst LD (2001). Highly expressed genes in yeast evolve slowly. Genetics.

[b53] Parmley JL, Hurst LD (2007). Exonic splicing regulatory elements skew synonymous codon usage near intron-exon boundaries in mammals. Molecular Biology and Evolution.

[b54] Plotkin JB, Kudla G (2011). Synonymous but not the same: the causes and consequences of codon bias. Nature Reviews Genetics.

[b55] Qin Z, Cai Z, Xia G, Wang M (2013). Synonymous codon usage bias is correlative to intron number and shows disequilibrium among exons in plants. BMC Genomics.

[b56] Rocha EP (2004). Codon usage bias from tRNA's point of view: redundancy, specialization, and efficient decoding for translation optimization. Genome Research.

[b57] Rodriguez O, Singh BK, Severson DW, Behura SK (2012). Translational selection of genes coding for perfectly conserved proteins among three mosquito vectors. Infection, Genetics and Evolution.

[b58] Rodríguez-Trelles F, Tarrío R, Ayala FJ (2006a). Origins and evolution of spliceosomal introns. Annual Review of Genetics.

[b59] Rodríguez-Trelles F, Tarrío R, Ayala FJ (2006b). Models of spliceosomal intron proliferation in the face of widespread ectopic expression. Gene.

[b60] Rogozin IB, Carmel L, Csuros M, Koonin EV (2012). Origin and evolution of spliceosomal introns. Biology Direct.

[b61] Roy SW, Gilbert W (2005a). Rates of intron loss and gain: implications for early eukaryotic evolution. Proceedings of the National Academy of Sciences of the United States of America.

[b62] Roy SW, Gilbert W (2005b). Resolution of a deep animal divergence by the pattern of intron conservation. Proceedings of the National Academy of Sciences of the United States of America.

[b63] Roy SW, Gilbert W (2005c). Complex early genes. Proceedings of the National Academy of Sciences of the United States of America.

[b64] Roy SW, Gilbert W (2005d). The pattern of intron loss. Proceedings of the National Academy of Sciences of the United States of America.

[b65] Roy SW, Gilbert W (2006). The evolution of spliceosomal introns: patterns, puzzles and progress. Nature Reviews Genetics.

[b66] Schneider DS, James AA (2006). Bridging the gaps in vector biology. Workshop on the molecular and population biology of mosquitoes and other disease vectors. EMBO Reports.

[b67] Severson DW, Behura SK (2012). Mosquito genomics: progress and challenges. Annual Review of Entomology.

[b68] Shabalina SA, Ogurtsov AY, Spiridonov AN, Novichkov PS, Spiridonov NA, Koonin EV (2010). Distinct patterns of expression and evolution of intronless and intron-containing mammalian genes. Molecular Biology and Evolution.

[b69] Subramanian S (2008). Nearly neutrality and the evolution of codon usage bias in eukaryotic genomes. Genetics.

[b70] Subramanian S, Kumar S (2004). Gene expression intensity shapes evolutionary rates of the proteins encoded by the vertebrate genome. Genetics.

[b71] Tripet F (2009). Ecological immunology of mosquito-malaria interactions: of non-natural versus natural model systems and their inferences. Parasitology.

[b72] Vinogradov AE (1999). Intron-genome size relationship on a large evolutionary scale. Journal of Molecular Evolution.

[b73] Vinogradov AE (2001). Intron length and codon usage. Journal of Molecular Evolution.

[b74] Wan XF, Xu D, Kleinhofs A, Zhou J (2004). Quantitative relationship between synonymous codon usage bias and GC composition across unicellular genomes. BMC Evolutionary Biology.

[b75] Warnecke T, Hurst LD (2007). Evidence for a trade-off between translational efficiency and splicing regulation in determining synonymous codon usage in *Drosophila melanogaster*. Molecular Biology and Evolution.

[b76] Waterhouse RM, Kriventseva EV, Meister S, Xi Z, Alvarez KS, Bartholomay LC, Barillas-Mury C (2007). Evolutionary dynamics of immune-related genes and pathways in disease-vector mosquitoes. Science.

[b77] Waterhouse RM, Wyder S, Zdobnov EM (2008). The *Aedes aegypti* genome: a comparative perspective. Insect Molecular Biology.

[b78] Yang YF, Zhu T, Niu DK (2013). Association of intron loss with high mutation rate in Arabidopsis: implications for genome size evolution. Genome Biology and Evolution.

[b79] Zhang L, Li WH (2004). Mammalian housekeeping genes evolve more slowly than tissue-specific genes. Molecular Biology and Evolution.

[b80] Zhu T, Niu D (2013). Frequency of intron loss correlates with processed pseudogene abundance: a novel strategy to test the reverse transcriptase model of intron loss. BMC Biology.

