# Supplementary material for: Antagonistic relationships between intron content and codon usage bias of genes in three mosquito species: functional and evolutionary implications
Source: Evol Appl. 2013 Jul 24;6(7):1079–89. doi: 10.1111/eva.12088 (PMC3804240; doi:10.1111/eva.12088)
Supplement: Supplementary file 1 [file eva0006-1079-SD1.doc]

**Table S4**. Summary results of the continuous regression tests. The beta parameter is the directional change parameter of Model B. It is interpreted as a regression coefficient of the trait values against total path length of phylogeny. Trait 1 represent intron data and trait 2 represent codon bias data. Variance (var) of both traits and corresponding covariance are shown for each tree.

| Tree No | Beta Trait 1 | Beta Trait 2 | Trait 1 Var | Trait 2 Var | Covariance |
| --- | --- | --- | --- | --- | --- |
| 1 | 0.189592 | -0.07784 | 27.28486 | 0.023792 | -0.24952 |
| 2 | 0.216773 | -0.3089 | 14.85181 | 0.033373 | -0.70193 |
| 3 | 0.218446 | -0.31508 | 16.02904 | 0.030572 | -0.69687 |
| 4 | 0.242403 | -0.53231 | 21.12938 | 0.028992 | -0.76191 |
| 5 | 0.20901 | -0.21039 | 11.34279 | 0.030242 | -0.55797 |
| 6 | 0.199203 | -0.14222 | 10.43755 | 0.030896 | -0.56557 |
| 7 | 0.201682 | -0.16141 | 10.77351 | 0.030621 | -0.57178 |
| 8 | 0.21853 | -0.29721 | 13.95026 | 0.029311 | -0.63861 |
| 9 | 0.209653 | -0.23035 | 12.97043 | 0.032004 | -0.61734 |
| 10 | 0.208517 | -0.21838 | 11.19856 | 0.031842 | -0.59331 |
| 11 | 0.200266 | -0.13587 | 8.65529 | 0.031031 | -0.51547 |
| 12 | 0.193806 | -0.06432 | 7.179087 | 0.03196 | -0.43993 |
| 13 | 0.203553 | -0.1524 | 8.303775 | 0.032899 | -0.49263 |
| 14 | 0.197127 | -0.09131 | 6.198284 | 0.032704 | -0.44142 |
| 15 | 0.20493 | -0.20048 | 12.40999 | 0.03274 | -0.63651 |
| 16 | 0.200425 | -0.14891 | 9.86998 | 0.032776 | -0.56788 |
| 17 | 0.196631 | -0.12274 | 11.12446 | 0.03127 | -0.56035 |
| 18 | 0.197128 | -0.12198 | 11.42491 | 0.029637 | -0.54521 |
| 19 | 0.198784 | -0.14552 | 10.88569 | 0.03276 | -0.59598 |
| 20 | 0.20126 | -0.15742 | 9.864261 | 0.033182 | -0.57188 |
| 21 | 0.202216 | -0.15924 | 10.21161 | 0.031397 | -0.56392 |
| 22 | 0.217025 | -0.30091 | 16.0431 | 0.028329 | -0.67413 |
| 23 | 0.207552 | -0.1906 | 9.149982 | 0.03268 | -0.54588 |
| 24 | 0.189793 | -0.03598 | 5.267883 | 0.033514 | -0.41461 |
| 25 | 0.19226 | -0.07686 | 8.096045 | 0.029654 | -0.48682 |
| 26 | 0.190439 | -0.05612 | 7.309042 | 0.029954 | -0.46038 |
| 27 | 0.199094 | -0.13592 | 10.014 | 0.031407 | -0.5476 |
| 28 | 0.207489 | -0.19938 | 12.06129 | 0.030944 | -0.57057 |
| 29 | 0.21508 | -0.2658 | 13.31161 | 0.031477 | -0.60488 |
| 30 | 0.21508 | -0.26851 | 13.4475 | 0.031799 | -0.61105 |
| 31 | 0.194395 | -0.09443 | 10.49609 | 0.028868 | -0.52706 |
| 32 | 0.194999 | -0.08942 | 10.03998 | 0.0312 | -0.51117 |
| 33 | 0.187084 | -0.01554 | 5.070215 | 0.033587 | -0.40353 |
| 34 | 0.189515 | -0.04003 | 6.20961 | 0.033463 | -0.4466 |
| 35 | 0.188331 | -0.0448 | 7.006188 | 0.032857 | -0.47952 |
| 36 | 0.190453 | -0.06112 | 7.46236 | 0.031126 | -0.47853 |
| 37 | 0.188255 | -0.03728 | 6.209459 | 0.033952 | -0.45861 |
| 38 | 0.186263 | -0.00612 | 4.742282 | 0.033106 | -0.39424 |
| 39 | 0.190454 | -0.05708 | 7.192467 | 0.031365 | -0.47208 |
| 40 | 0.187981 | -0.02865 | 6.088236 | 0.030503 | -0.42482 |
| 41 | 0.191405 | -0.07735 | 8.696201 | 0.030501 | -0.514 |
| 42 | 0.189118 | -0.05663 | 7.81322 | 0.031138 | -0.49254 |
| 43 | 0.186011 | -0.02833 | 6.972352 | 0.031891 | -0.47114 |
| 44 | 0.200524 | -0.14041 | 8.65085 | 0.033533 | -0.53398 |
| 45 | 0.2466 | -0.57475 | 24.60597 | 0.026035 | -0.79062 |
| 46 | 0.234756 | -0.44722 | 14.18895 | 0.033709 | -0.6804 |
| 47 | 0.254834 | -0.59416 | 18.63931 | 0.028597 | -0.7137 |
| 48 | 0.219367 | -0.29276 | 14.07895 | 0.030806 | -0.59166 |
| 49 | 0.213308 | -0.26189 | 14.75055 | 0.032526 | -0.63233 |
| 50 | 0.195784 | -0.09354 | 9.963251 | 0.030818 | -0.50191 |
| 51 | 0.188365 | -0.06128 | 9.601591 | 0.030916 | -0.5406 |
| 52 | 0.19121 | -0.07823 | 9.36779 | 0.028474 | -0.51163 |
| 53 | 0.190582 | -0.07032 | 8.706373 | 0.02879 | -0.49712 |
| 54 | 0.189285 | -0.06803 | 10.18585 | 0.031162 | -0.55203 |
| 55 | 0.192284 | -0.08743 | 9.828445 | 0.028771 | -0.52448 |
| 56 | 0.189644 | -0.05845 | 7.916278 | 0.030768 | -0.49037 |
| 57 | 0.188364 | -0.04869 | 7.009503 | 0.033516 | -0.47925 |
| 58 | 0.183586 | 0.001834 | 4.731231 | 0.032448 | -0.3885 |
| 59 | 0.187447 | -0.04408 | 7.219514 | 0.03073 | -0.46703 |
| 60 | 0.187505 | -0.04374 | 7.33613 | 0.031901 | -0.47844 |
| 61 | 0.196356 | -0.11078 | 7.890686 | 0.033968 | -0.51529 |
| 62 | 0.195872 | -0.10247 | 8.382228 | 0.031017 | -0.50646 |
| 63 | 0.193202 | -0.08349 | 8.104224 | 0.032096 | -0.50852 |
| 64 | 0.191342 | -0.05766 | 8.035257 | 0.029859 | -0.45028 |
| 65 | 0.196393 | -0.12907 | 11.3113 | 0.031744 | -0.59898 |
| 66 | 0.19482 | -0.10376 | 11.90806 | 0.030142 | -0.54176 |
| 67 | 0.187131 | -0.0352 | 7.127107 | 0.031914 | -0.4619 |
| 68 | 0.188116 | -0.05867 | 9.022314 | 0.029548 | -0.5109 |
| 69 | 0.186498 | -0.03674 | 6.319162 | 0.031695 | -0.44708 |
| 70 | 0.184149 | -0.0324 | 7.809126 | 0.029487 | -0.4794 |
| 71 | 0.183267 | 0.003127 | 4.419384 | 0.03348 | -0.38458 |
| 72 | 0.183842 | -0.0074 | 5.105696 | 0.033025 | -0.4102 |
| 73 | 0.184873 | -0.00887 | 4.863085 | 0.033544 | -0.39806 |
| 74 | 0.183377 | -0.00983 | 5.527616 | 0.03275 | -0.42424 |
| 75 | 0.18312 | -0.00147 | 4.73082 | 0.033055 | -0.39431 |
| 76 | 0.183414 | -0.00809 | 4.862887 | 0.03123 | -0.3892 |
| 77 | 0.186622 | -0.04446 | 7.265425 | 0.031849 | -0.48027 |
| 78 | 0.186237 | -0.03512 | 7.190433 | 0.033004 | -0.48628 |
| 79 | 0.186326 | -0.01494 | 5.381793 | 0.033274 | -0.42316 |
| 80 | 0.188985 | -0.04917 | 6.436306 | 0.03437 | -0.47032 |
| 81 | 0.192971 | -0.08425 | 8.091716 | 0.032751 | -0.50946 |
| 82 | 0.192345 | -0.07056 | 6.92454 | 0.032255 | -0.46983 |
| 83 | 0.19161 | -0.07582 | 8.158158 | 0.032357 | -0.51191 |
| 84 | 0.189605 | -0.04326 | 6.123751 | 0.032237 | -0.43285 |
| 85 | 0.188678 | -0.05077 | 7.123536 | 0.030227 | -0.45545 |
| 86 | 0.184315 | -0.0143 | 5.736215 | 0.031213 | -0.42313 |
| 87 | 0.184276 | -0.02038 | 6.300389 | 0.030978 | -0.43588 |
| 88 | 0.183669 | -0.01562 | 6.254026 | 0.031364 | -0.43721 |
| 89 | 0.183448 | -0.00952 | 6.562828 | 0.031952 | -0.44063 |
| 90 | 0.185429 | -0.02429 | 6.760048 | 0.030365 | -0.44127 |
| 91 | 0.185716 | -0.01641 | 5.234756 | 0.030561 | -0.39986 |
| 92 | 0.18801 | -0.03705 | 6.50342 | 0.033548 | -0.46658 |
| 93 | 0.188873 | -0.04284 | 6.269166 | 0.034113 | -0.46161 |
| 94 | 0.18776 | -0.04264 | 6.491179 | 0.033771 | -0.46792 |
| 95 | 0.183705 | -0.01024 | 6.017785 | 0.034731 | -0.45699 |
| 96 | 0.184505 | 0.003133 | 4.77631 | 0.032005 | -0.37654 |
| 97 | 0.190954 | -0.09114 | 10.26539 | 0.030736 | -0.55925 |
| 98 | 0.186879 | -0.05786 | 8.986557 | 0.030374 | -0.52081 |
| 99 | 0.184938 | -0.04301 | 8.612414 | 0.030639 | -0.513 |
| 100 | 0.183235 | -0.01707 | 6.766992 | 0.034178 | -0.47509 |
