# Supplementary material for: Antagonistic relationships between intron content and codon usage bias of genes in three mosquito species: functional and evolutionary implications
Source: Evol Appl. 2013 Jul 24;6(7):1079–89. doi: 10.1111/eva.12088 (PMC3804240; doi:10.1111/eva.12088)
Supplement: Supplementary file 2 [file eva0006-1079-SD2.docx]

Table S2. Canonical correlations between synonymous codon usage orders (SCUO) and intron contents of 1:1:1 orthologous genes among three mosquito species.

_________________________________________________________________________

Intron^#^  Correlation^*^ Eigenvalue Test_statistics^**^ Significance

_________________________________________________________________________

1 -0.213 0.36503 0.133245 *p* = 0.058

2 -0.119 0.25724 0.066174 *p* = 0.242

3 -0.079 0.24429 0.059679 *p* = 0.306

__________________________________________________________________________

^#^ Different measures of intron contents: 1) counts of introns, 2) intron length normalized by total amount of coding sequences, and 3) intron length normalized by gene length.

^*^ Mean correlation of the principal canonical axis with variation between codon bias and intron content.

^**^ Permutation test (first squared canonical correlation).
