# Supplementary material for: Antagonistic relationships between intron content and codon usage bias of genes in three mosquito species: functional and evolutionary implications
Source: Evol Appl. 2013 Jul 24;6(7):1079–89. doi: 10.1111/eva.12088 (PMC3804240; doi:10.1111/eva.12088)
Supplement: Supplementary file 3 [file eva0006-1079-SD3.docx]

Table S3. Results of pair-wise *a posteriori* comparison between gene groups (see Figure 3). These genes vary in intron content and codon biases of the genes are compared between groups. The multivariate *t*-statistic (based on Euclidean distance) and significance (*p*-value) are shown.

| **Group pair** | ***t*- statistics** | ***p*- value** |
| --- | --- | --- |
| g1 - g2 | 2.6153 | 0.005 |
| g1 - g3 | 0.3443 | 0.9301 |
| g1 - g4 | 2.0638 | 0.022 |
| g1 - g5 | 1.1315 | 0.2637 |
| g1 - g6 | 1.9736 | 0.028 |
| g2 - g3 | 2.8688 | 0.001 |
| g2 - g4 | 2.4867 | 0.005 |
| g2 - g5 | 1.6212 | 0.0919 |
| g2 - g6 | 0.9528 | 0.3896 |
| g3 - g4 | 2.4168 | 0.001 |
| g3 - g5 | 1.2547 | 0.2158 |
| g3 - g6 | 2.2719 | 0.008 |
| g4 - g5 | 1.695 | 0.0599 |
| g4 - g6 | 2.2726 | 0.009 |
| g5 - g6 | 1.2446 | 0.1978 |
