# Supplementary figures and images for: Antagonistic relationships between intron content and codon usage bias of genes in three mosquito species: functional and evolutionary implications
Source: Evol Appl. 2013 Jul 24;6(7):1079–89. doi: 10.1111/eva.12088 (PMC3804240; doi:10.1111/eva.12088)

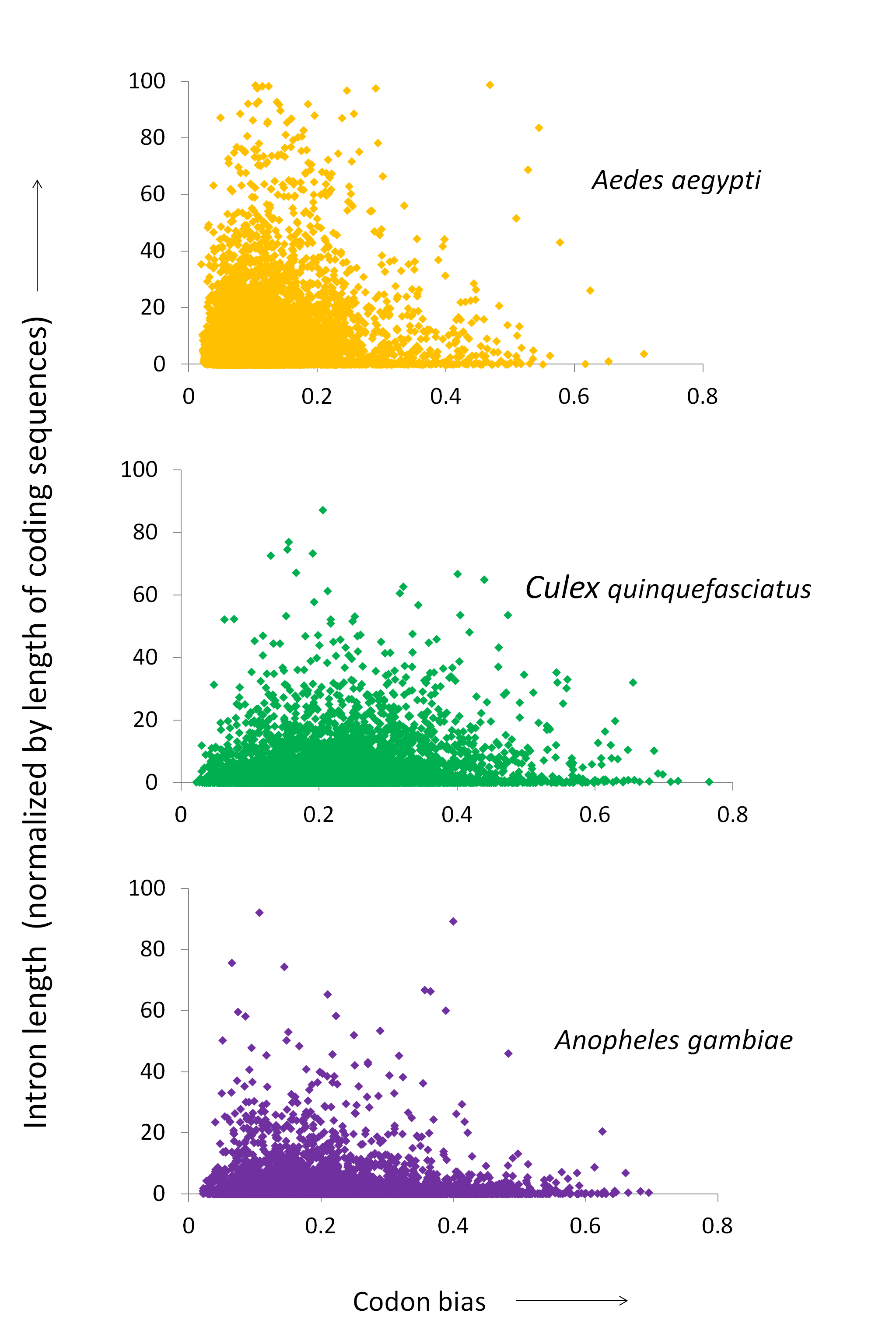

Supplement: Supplementary file 4 [file eva0006-1079-SD4.tif]
